# Major Thomas Alfred Perry Marsh

**Published:** 1900-06

**Authors:** 


### Obituary.

Major Thomas Alfred Perry Marsh, R.A.M.C., whose death was reported at Deelfontein Hospital on 22nd May, 1900, was the youngest son of the late Captain Henry Godfrey Marsh, J.P., of Winterbourne Park, Gloucestershire.

He was born at Blackwood, Mon., on the 23rd February, 1856, and received his professional education at University College, Bristol, and St. George's Hospital, London, and he was admitted a member of the Royal College of Surgeons, England, 1881; Lic. Royal College of Physicians, London, 1882; D.P.H., R.C.P.S., London, 1892. Major Marsh was elected a Fellow of the Royal Institute of Public Health in 1899.

At the University College, Bristol, he had a brilliant career, where he was the first and third year's Prizeman, Clark Scholar in Surgery, Sanders Scholar in Medicine, and Haberfield Prizeman in Medicine, Surgery, and Obstetrics. He held for a short time the house-surgeoncy at Weston-super-Mare Hospital. In July, 1882, he passed high in the list as probationary surgeon to Netley, and became Surgeon-Major in 1894.

Major Marsh saw active service in the Burmese Expedition in 1885 to 1887, when he was in medical charge of a battery of mountain artillery and was present in several engagements. For this campaign Major Marsh had the medal with clasp.

He was ordered to South Africa in October, 1899, in special charge of No. 3 Stationary Field Hospital, of which he himself personally superintended the equipment, and sailed for South Africa in the "Kildonan Castle" on 4th of November, 1899. His hospital was subsequently stationed at the important junction of De Aar, where at the time of his death he was in command, and also P.M.O. for this important camp and district, and at this place his duties became at once very onerous, and, as he described it, his hospital grew as requirements demanded from 100 to 500 beds, and before he could get accommodation complete he was inundated with the wounded from Graspan, Modder River, and Magersfontein, all of which actions were fought within a short time of his arrival at De Aar. As Lord Roberts invaded the Free State his hospital became crowded with enteric fever, and in one of his late letters home he said he then had 200 cases under treatment. With these onerous duties, never having quite recovered the exposure and privation of the Burmese campaign, from whence he was invalided home, his health gave way. He was sent from De Aar to recoup at the hospital of the Imperial Yeomanry at Deelfontein, but it was there found that he had contracted the disease (enteric fever) which he had been combating, and to which, to the intense sorrow of all his many friends, he succumbed on 22nd May, 1900—truly a life given in noble devotion to his duty and country.

Major Marsh was a fluent speaker and a brilliant conversationalist. He was the author of "Magazine Rifles in War." This was given as a paper at the United Service Institute, and considered of such merit that the Institute afterwards published it in pamphlet form. He wrote also on "Antipyretics and the Rational Treatment of Fevers," and "Young Soldiers in India."

Major Marsh married Miss Cecilia Bacquera, daughter of Señor Don Vincent Bacquera, of Malaga, Spain, and great sympathy is felt for his young widow in her bereavement. His untimely death, at the age of 44, has cut short a brilliant career, and is an intense grief to his relatives and his large circle of friends.

